# Evaluating pre-anesthesia assessment performance in residency: the reliability of standardized patient methods

**DOI:** 10.3389/fmed.2024.1342004

**Published:** 2024-08-01

**Authors:** Emmanuel Besnier, Sébastien Franchina, Antoine Lefevre-Scelles, Thierry Wable, Jean-Luc Hanouz, Etienne Allard, Bertrand Dureuil, Vincent Compère

**Affiliations:** ^1^Department of Anesthesia and Intensive Care, Rouen University Hospital, Rouen, France; ^2^Normandie Univ, UNIROUEN, Inserm, Mont-Saint-Aignan, France; ^3^UFR Médecine Pharmacie, Université de Normandie, Rouen, France; ^4^Department of Anesthesia and Intensive Care, Caen University Hospital, Caen, France; ^5^Department of Anesthesia, Le Havre Hospital, Le Havre, France

**Keywords:** simulation, preanesthesia assessment, simulated patients, anesthesia, consultation

## Abstract

**Background:**

Pre-anesthesia assessment clinic (PAC) is known to increase safety and quality in the perioperative period. However, PAC teaching during anesthesiology residency is a challenge. The objective of this study was to assess the reliability of a simulation score grid using a standardized patient on the PAC performance of anesthesiology residents.

**Methods:**

A score grid, including the 4 components of the PAC (clinical evaluation, perioperative strategy, information and communication) was validated by a group of 5 senior anesthesiologists. Anesthesiology residents (> one year) and attending anesthesiologists were included. The same simulation sequence with the same standardized patient was conducted in a simulation dedicated consultation room. The simulation sequence was followed by a debriefing session with the 2 professors (anesthesiology and communication) and each anesthesiology resident. The main outcome was the overall grid score out of a maximum score of 300 and the correlation of this score with experience in anesthesiology residency. Secondary outcomes were individual component scores according to level of experience in anesthesiology.

**Results:**

Between October 2014 and April 2016, 109 anesthesiology residents and 16 attending anesthesiologists were included in this prospective bicentric study. There was a positive correlation (*p* < 0.01) between level of experience and overall score on the grid score (Pearson’s Coefficient = 0.52). The Pearson correlation coefficient between overall assessment and level of experience in anesthesiology was 0.46 (*p* < 0.01). The analysis of the sub-scores for the 4 components of the overall score (evaluation, perioperative strategy, information and communication) also identify differences between groups of experience.

**Conclusion:**

Standardized patient Simulation of PAC seems to be a reliable tool to assess PAC performance in anesthesiology residents and senior anesthesiologists. These results suggest standardized patient simulation could be used as a teaching tool for PAC.

## Highlights

- Question: Is simulation tool using a standardized patient on the preanesthesia consultation assessing the performance of anesthesiology residents?
- Finding: There was a positive correlation (*p* < 0.01) between level of resident’s experience and overall score on the grid score (Pearson’s Coefficient = 0.52).
- Meaning: Standardized patient simulation of PAC seems to be a reliable tool to assess PAC performance in anesthesiology residents and attending anesthesiologists.

## 1 Introduction

Pre-anesthesia Assessment Clinic (PAC) has been developed to assess preoperative risks related to surgery or patient illness, to select laboratory tests, and to consider the best perioperative strategy and anesthesia techniques. Some guidelines have been edited to ensure quality and reliability of this assessment (1, 2). PAC is associated with lower perioperative morbi-mortality (3, 4), preoperative optimization of patients (5), lower costs related to fewer surgical cancelations or reduced length of stay (6) and lower costs related to fewer preoperative tests (7). Conversely, inadequate PAC can lead to incidents during and after surgery (8). In addition, PAC is an appropriate time to educate patients on anesthesia, perioperative care and pain treatments, to reduce anxiety, to develop care plans and to obtain informed consent (9, 10).

The PAC could be divided in two stages (1) an interview about the patient’s medical, anesthetic, surgical or allergic history and personal medication with a physical evaluation including venous or upper airway access, and (2) a discussion about adequate perioperative management and medical risks to obtain the patient’s informed consent. PAC is a difficult exercise and requires different physician skills including medical but also non-technical skills as communication or organization (11). Preoperative Assessment Clinic (PAC) is indeed a complex exercise that necessitates a wide range of physician skills, including not only medical expertise but also non-technical skills such as communication and organization (11). At each stage of the PAC process, unique challenges arise that demand specific skills to ensure optimal patient outcomes (12). During the initial patient evaluation, effective communication skills are crucial in obtaining an accurate medical history and understanding the patient’s concerns and expectations (13). Physicians must be able to actively listen, ask pertinent questions, and provide clear, concise explanations to establish trust and rapport with the patient. Poor communication at this stage can lead to misunderstandings, misdiagnosis, and inappropriate treatment plans. The next stage involves preoperative testing and risk assessment, where organizational skills come into play. Physicians must efficiently coordinate various diagnostic tests and consultations, ensuring that all necessary information is obtained in a timely manner. They must also be adept at interpreting test results and assessing the patient’s risk profile to make informed decisions regarding perioperative management. Challenges at this stage may include managing time constraints, dealing with incomplete or conflicting data, and navigating complex medical conditions. In the preoperative optimization phase, medical expertise is paramount. Physicians must be knowledgeable about various medical conditions and their potential impact on surgical outcomes (4). They must also be skilled in prescribing appropriate interventions to optimize the patient’s medical status and minimize perioperative risk. Challenges at this stage may include managing co-morbidities, balancing the risks and benefits of various interventions, and dealing with patient non-compliance. Finally, the perioperative planning stage requires a combination of medical, communication, and organizational skills. Physicians must effectively communicate their findings and recommendations to the surgical team, coordinate care with other healthcare providers, and develop a comprehensive perioperative plan that addresses the patient’s unique needs and preferences (14). Challenges at this stage may include navigating interdisciplinary dynamics, managing conflicting opinions, and ensuring that all relevant information is accurately documented and communicated.

PAC learning during anesthesia residency is not well defined regarding either its objectives or its practical realization. Moreover, PAC teaching involves self-learning with or without oral guidance by senior anesthesiologists based on their own experience. Published data about teaching are scarce and the interest of one existing report on problem-based learning for PAC is not clear (15). We previously conducted a French national survey on anesthesiology residents’ and teachers’ opinions of PAC teaching (16). Residents described PAC as a major and difficult act performed mostly alone and considered PAC teaching in France to be insufficient; teachers considered that PAC teaching could be improved using simulation.

Based on the approach of the objective structured clinical examination (OSCE) (17), standardized patient simulation score grid could be a reliable tool to evaluate pre-anesthesia assessment performance (18). Several studies have explored various aspects of validity of the Objective Structured Clinical Examination (OSCE), including content, response process, internal structure, relations with other variables, and consequences. Hodges et al. showed that the test items are representative of the skills and knowledge being assessed (19). The response process was investigated by Daniels et al. who focused on the coherence and consistency of the data collected during the examination (20). The consequences of OSCEs on learners, instructors, and the curriculum are well described, highlighting the impact and implications of these examinations in medical education (21). This kind of tool has been described to be a valuable learning approach for assessment of medical students and can be used for formative assessment by association of a personalized debriefing session (22). OSCE has also been described as a valuable and reliable tool for communication assessment (23–25). In this work, we describe the development and the evaluation of standardized patient simulation score grid for PAC. The main objective of this study was to assess the reliability of this tool on the PAC performance of anesthesiology residents.

## 2 Materials and methods

### 2.1 Ethics statement

This research project has received the approval of the Ethics Committee for Non-Interventional Research of Rouen University Hospital (N° E2014-18). The requirement for written informed consent was waived by the Committee. All participants were informed beforehand of the principle of the simulation session and its objectives, as well as the potential interest for training. The presence of an audio-visual system for the real-time retransmission of the session without recording or image processing as well as the presence of professors was reported. All participants have given oral agreement to participate. In the event of occasional image retention, written consent for the image has been signed by each participant.

### 2.2 Description of the study

Based on the principle of OSCE, we developed a standardized patient simulation adapted to PAC. There were not several successive stations but only one, with an overall duration of 20 to 30 min. This choice was made so as not to artificially fragment the individual components of this consultation which are in common practice all interdependent. We followed the Association for Medical Education in Europe (AMEE) guidelines for the development of the tool, the scenario, the scorecard and for the evaluation of this tool (22).

### 2.3 Population

We conducted a prospective bicentric study, including anesthesiology residents (128 students eligible) and seniors (16 seniors on a voluntary basis) from Rouen and Caen University Hospitals. All participants had the same session, in the same place, with the same simulated and standardized patient. No previous specific training had been dedicated to consultation.

### 2.4 Conducting the sessions

Each participant took part in the evaluation only once. Participants were individually summoned to the medical office of Rouen University’s school of medicine, comprising 2 adjoining rooms:

– A standard clinical room with a desk, a computer (non-functioning), a telephone, basic clinical examination equipment (examination table, stethoscope, blood pressure monitor) and realistic decorations as well as blank paper supports. The specificity of this room is the presence of a high-performance microphone fixed to the ceiling above the desk and a very high definition-rotating camera fixed to the wall allowing an overall view of the room.
– Another room with a 120 cm flat TV screen fixed to the wall, a telephone and an instant audio-visual broadcasting system via the TV screen and 2 loudspeakers fixed to the ceiling. This system allows real-time transmission from the adjoining clinical room. It also allows the recording and processing of images and sound, only when a single computer with dedicated software is connected.

A short fact sheet recalling the clinical context was placed in the clinical room. The instruction given was to perform PAC as usual. Then the participant went to fetch the standardized patient who was waiting in the corridor and conducted PAC using the information provided. A maximum of 20 min was allowed. A neutral paper support was given. The intervention of observers during PAC was allowed in a strictly exceptional manner and only in the event of significant blocking of the situation or of manifest delay.

During each simulation session, the same anesthesiology (VC) and communication (TW) professors observed the sequence and filled an evaluation form with a dedicated score grid with a maximum of 300 points.

A subjective qualitative evaluation of the session represented by the letters A to E (A = very good, B = good, C = average, D = insufficient, E = extremely insufficient), according to the simulated patient’s report (the same female for all the cohort). Immediately after the PAC session and before any comment or other communication, in order to avoid any influence, the simulated patient entered his/her subjective notation in the grid and then reported back to the observers in the absence of the participant.

Finally, the anesthesiology and the communication professors carried out a 10–15 min personalized debriefing session with each resident highlighting the medical, behavioral, communication or information components, in a voluntarily positive and advisory spirit, avoiding any judgment.

### 2.5 Construction of the standardized scenario and simulated patient training

We constructed a moderately difficult standardized scenario based on real facts, accessible even to inexperienced young residents. The standardized patient was a female patient scheduled for knee arthroscopy and with clinically predictive criteria for difficult ventilation and intubation. This case scenario was associated with paper supports (prescription, laboratory examinations or specialized consultation reports) in order to increase immersion in the scenario. We recruited one standardized volunteer patient, unpaid, naive of medical skills and totally unknown to the participants in the simulation. She received a precise presentation of the work and the sessions, an explanation of the scenario and the information necessary to assimilate the proposed situation, as well as instructions on desired behavior that could be modulated. A single standardized patient was recruited and trained for this scenario, in order to conserve reproducibility of sessions and homogeneity of responses, because the same situation was proposed to all participants.

### 2.6 Construction of the score grid

Using the quality criteria described above (22), we developed a binary (yes-no) rating grid, subdivided into 4 parts, corresponding to the 4 main objectives of PAC: Evaluation / Strategy / Information / Communication. It consisted of 83 positive items (evaluation = 41, strategy = 17, information = 20, communication = 5), the weighting of which was adjusted according to the *a priori* importance of each item and the grid contained 6 negative items on major elements or guidelines. The maximum score for this grid was 300 points (evaluation = 116, strategy = 72, information = 59, communication = 53, maximum negative points = −160). The minimum score was 0 even in the case of a negative score. This score grid was validated by a consensus of 5 anesthesiologists (2 professors of anesthesiology and 3 seniors anesthesiologists).

### 2.7 Satisfaction survey of residents

Following each session of PAC simulation with personalized debriefing, an e-mail satisfaction survey was sent to all participating anesthesiology residents. This self-questionnaire was edited using Google Forms© software. It consisted of 10 multiple choice questions and required 3 min to answer.

### 2.8 Data collected

The data collected for each participant during these simulation sessions were:

– Total and partial score (based on the 4 PAC components) on the score grid.
– Rating of the qualitative assessment (A B C D or E) by simulated patient.
– The level of experience in anesthesiology of each participant.
– Participants’ opinions on the value of PAC simulation for assessment/training in anesthesia consultation.

### 2.9 Study outcomes

The primary outcome was the performance assessed by the grid score according to the experience of anesthesiology residents.

Secondary outcomes included:

– Results for each component (evaluation, strategy, information, communication) according to the experience in anesthesiology.
– Measurement of the 7 applicable criteria of validity and reliability of session according to the AMEE (22).
– Satisfaction of the simulation session from anesthesiology residents on a 10 points scale.

### 2.10 Statistical analysis

It was not possible to calculate an *a priori* number of subjects required because of the pilot nature of this study. This was arbitrarily set at a minimum of 100 subjects with a minimum of 15 residents per year (5 years to validate the French anesthesiology residency). Data are presented as median and interquartile range. Because the distribution of the population was normal as explored using a Shapiro–Wilk test, an Analysis of Variance (ANOVA one-way) test was used for quantitative data. In case of significant results for the overall test, a post-test was performed to explore differences between groups (Turkey’s multiple analyses). Qualitative data were compared using a Chi^2^ test. The correlation between experience of residents in years was correlated with the overall grid score using a Pearson’s correlation test, and r value were presented with 95% confidence intervals. *p* < 0.05 was considered significant for all these analyses. Because experience of the seniors may be heterogeneous, we did not correlate their experience with their overall scores. Finally, the scoring grid was subjected to the Cronbach alpha coefficient test to assess its internal validity. All analyses were performed using graphpad prism v8.0 (La Jola, USA).

## 3 Results

**Figure 1:**
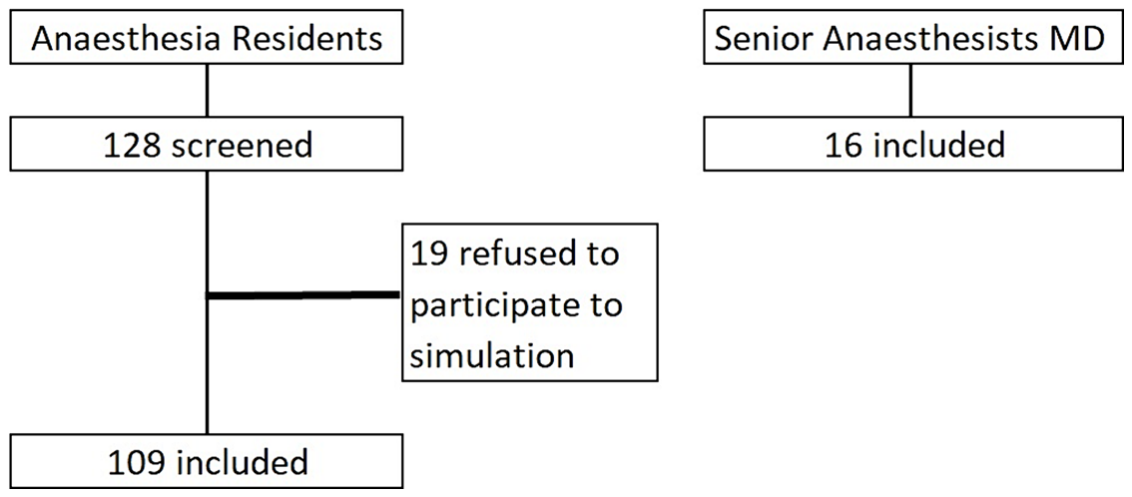
Flow chart diagram.

Between October 2014 and April 2016, 125 anesthesiologists (16 seniors and 109 residents were included), with 57% from Rouen University Hospital and 43% from Caen University Hospital (Figure 1). This geographical distribution ensures a diverse representation of anesthesiology training within the region.

**Figure 2:**
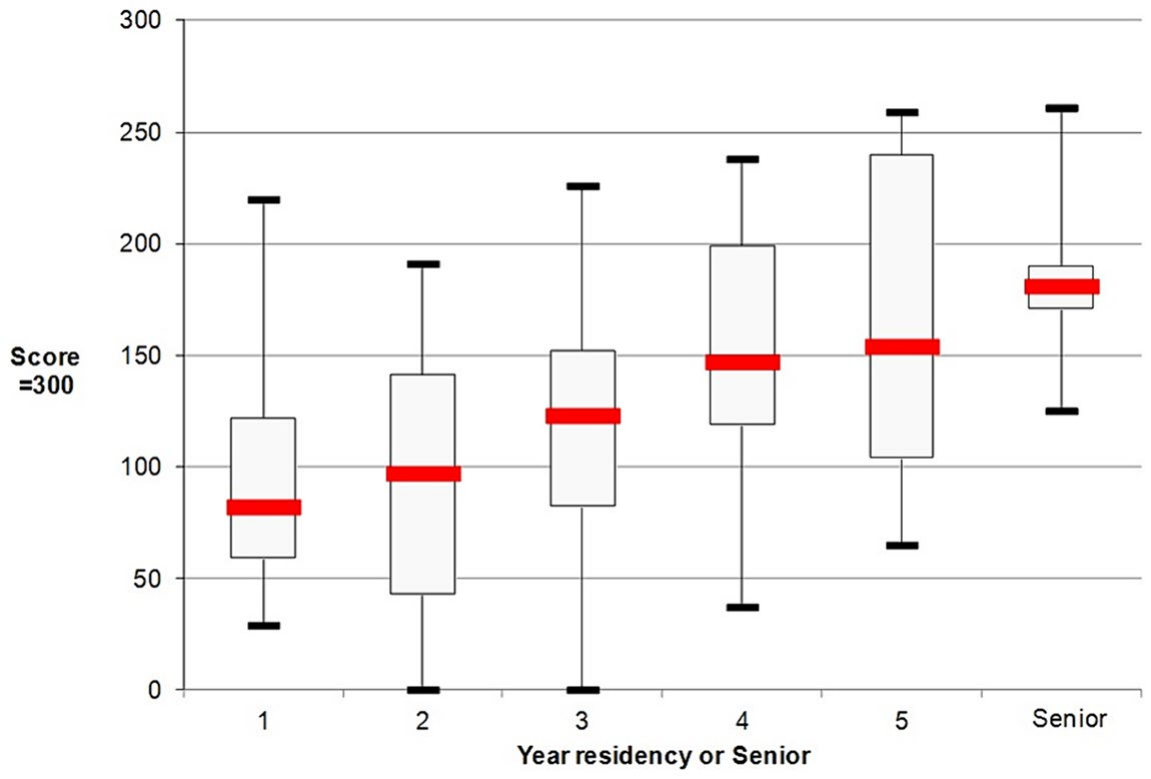
Grid score according to level of experience in anesthesiology. The score was presented with median and first-third interquartile and minimal and maximal scores according to level of experience in anesthesiology (one category for each of the 5 years of residency and a category of senior anesthesiologist). *First year versus others; †second year versus others; ‡third year versus others. No difference was observed for 4 and 5 years between each other and versus seniors. *p < 0.05; **p < 0.01; ****p < 0.0001. Analysis was performed using an ANOVA test with Dunn’s post-test.

The score is presented in Figure 2 according to the experience of anesthesiologist. There was a correlation between the score and the experience of the residents in years (r = 0.43 IC95% [0.26–0.57], *p* < 0.0001). The analysis of the sub-scores for the 4 components of the overall score (evaluation, perioperative strategy, information and communication) is presented in Table 1. According to AMEE 2010 guidelines, seven reliability criteria were analyzed (Table 2).

**Table 1:** Median scores with first and third quartiles for the 4 different components of the paranesthesia assessment grid according to clinical experience.

	Year 1 Residents (*n* = 27)	Year 2 Residents (*n* = 23)	Year 3 Residents (*n* = 21)	Year 4 Residents (*n* = 23)	Year 5 Residents (*n* = 15)	Seniors (*n* = 16)
Evaluation	73 [65–81]	72 [66–83]	78 [66–86]	86 [76–92]^*^	85 [74–97]^*^	91 [86–97]^****, †††, ‡^
Peri operative Strategy	36 [28–41]	35 [24–41]	39 [29–43]	42 [37–50]^*, †^	42 [36–54]^*, †^	47 [42–50]^**, ††^
Information	20 [14–27]	28 [17–31]	29 [25–34]^*^	34 [27–41]^****, †^	36 [32–44]^****, ††††, ‡‡^	41 [36–45]^****, ††††, ‡‡‡, #^
communication	35 [28–46]	37 [30–44]	40 [29–43]	44 [39–48]	41 [35–48]	50 [48–50]^****, ††††, ‡‡‡‡^

Maximum score for Evaluation: 116, perioperative strategy: 72, information: 59, communication: 53.*First year versus others; †second year versus others; ‡third year versus others; #fourth year versus others.*p < 0.05; **p < 0.01; ****p < 0.0001.Analysis was performed using an ANOVA test with Turkey’s post-test.

**Table 2:** Criteria of validity and reliability of OSCE according to the Association of Medical Education in Europe guidelines published in 2010 (26).

Criteria of validity and reliability according to AMEE 2010		Objective	Our study
Alpha coefficient of Cronbach on the score grid		> 0.7	0.752
Pearson coefficient interevaluation between the qualitative assessment by the simulated and standardized patient and the grid score		> 0.5	0.82
Pearson coefficient between evaluation and expertise	Performance evaluated by score grid	> 0.5	0.52
	Performance evaluated by overall qualitative assessment	> 0.5	0.46
Intergrade discrimination		≤ 10%	16%
Number of failures (evaluation D or E)		“low”	15%
Insufficient number of notations (<10/25) by the simulated patient		≤ 10%	9%

The satisfaction (70% response rate) for the session was 8 (8, 9) with a relevance of the training of 9 (8–10).

## 4 Discussion

The standardized patient (simulated patient) is a type of human simulation that uses a well-trained healthy person (actor) to play the role of the real patient with stimulating his physical condition wherein the trainees (students) can train on the medical skills (27). Our work shows that the performance of anesthesiology residents in PAC evaluated with a grid score in a context of standardized patient session is correlated with their level of experience in anesthesiology. This result is in agreement with several works published in other medical specialties. In general practice, Hodges et al. showed in 42 students and physicians, an increase in the evaluation according to experience (28). In the same field, Prislin et al. reported a good in 335 general medical students (29). In emergency medicine, Wallenstein et al. confirmed the validity of score grid to represent the performance of 239 students (30). Finally, in neurology, Lukas et al. in 195 students at the end of the curriculum, found a correlation between a grid score performance and results at the final exams (31).

Medical communication and patient information are main factors of satisfaction in anesthesia (32, 33). Bondy et al. showed that the information delivered during PAC allowed a reduction of anxiety in patients (34). Similarly, Soltner et al. suggested that the attitude of the anesthesiologist in consultation helped to reduce this anxiety (10). Regarding this relational aspect, simulation has already been used especially in geriatrics for formative evaluation focused on communication skills. Indeed, Lauren et al., with 19 students and Ishikawa et al. with 85 residents, suggested the tool’s usefulness for training in verbal or non-verbal communication skills (35, 36). Finally, O’Sullivan et al. proposed the use of OSCE and simulation for the assessment and training of relational skills (37). Similarly, in our work, information and communication seem to be the most difficult dimensions of CPA to grasp., especially by the youngest residents. The pivotal role of medical communication and patient information in enhancing patient satisfaction and reducing anxiety within anesthesia settings cannot be overstated. Effective communication skills and positive attitudes on the part of anesthesiologists can significantly alleviate patient anxiety and improve overall satisfaction with care (38, 39). This underscores the importance of fostering psychological well-being among medical professionals, as this can translate into more compassionate and effective patient care. A recent study highlighted the significance of psychological factors and their relationship with academic performance of veterinary students (40). This study suggests that psychological well-being not only impacts academic success but also has profound implications for professional interactions, including those in medical settings. By cultivating strong communication skills and empathetic attitudes, anesthesiologists can create a supportive and reassuring environment for patients, thereby enhancing their overall experience and reducing preoperative anxiety. The integration of psychological factors into our understanding of medical communication and patient satisfaction offers a more holistic perspective on the role of anesthesiologists in patient care. By recognizing the importance of psychological well-being and its impact on professional interactions, we can better appreciate the value of effective communication and empathetic attitudes in enhancing patient outcomes and improving the quality of care in anesthesia settings.

In our study, the level of satisfaction of the anesthesiology residents of the PAC simulation was high. This is in agreement with numerous data from the literature in different medical specialties (41–43). Specifically in anesthesia, Jindal et al. showed the good adhesion and satisfaction of the residents who had OSCE during their studies (44). Despite all this, feedback from our PAC simulation participants revealed a disruption in their classic consultation pattern, probably due to the neutral written support provided and not directive support. This choice was made so as not to introduce bias between the participants by using a preexisting anesthesia form and therefore potentially known to some of the participants. Also, PAC performance should not be dependent on the support used, which is in fact only a tool to help medical evaluation, and remains only one component among others during PAC. Simmonds et al. suggested a slight improvement in the completeness of this evaluation by providing directive support (45). Similarly in 2002, Ausset et al. about 964 retrospective records showed that the standardization of patient evaluation by a written guide improves the completeness of this evaluation without prejudging the impact on patient outcomes (46). However, our results show that the evaluation scores were rather low in the different groups and that the participants were not generally in difficulty on this point.

Although 125 anesthesiologists were included, the present study is not without limitations. First, our score grid was developed and validated by 5 senior anesthesiologists from the same university hospital which could introduce an evaluation bias. Nevertheless, the PAC components included in our grid are in line with those proposed by the ASA Task Force on Preoperative Evaluation or by the Brazilian teachers’ college, and those included in a literature review by Klafta and Roizen for information and relational aspects (1, 2, 47, 48). So our grid integrates collecting medical history, treatment and physical examination of the patient; targeting patient-adapted anesthetic strategy issues and information related to anesthesia; selecting additional tests to be performed depending on the field and surgery; inform the patient about the anesthetic process and its risks; obtaining informed consent and positive communication and interaction with the patient. On the other hand, our PAC simulation meets the majority of quality criteria published by AMEE except for inter-grade discrimination. This weakness is most likely related to the presence of a few very low scores which lead to a measurement bias and an increase in the linear regression slope between the two evaluation methods (the score grid versus overall qualitative evaluation). According to the AMEE, these low scores could be excluded from the analyses in order to avoid an excessive impact on the qualitative criteria of the tool. This makes it possible to moderate the negative impact of this criterion on the overall quality of our simulation. Finally, like all simulation tools, an important limit to the development and dissemination of OSCE is linked to the time, architecture or logistic preparation required for its implementation.

## 5 Conclusion

Standardized patient Simulation of PAC seems to be a reliable tool to assess PAC performance in anesthesiology residents and senior anesthesiologists. These results suggest standardized patient simulation could be used as a teaching tool for PAC. We used a single scenario. The grid can of course be adapted to other clinical cases. This work proposes a grid that can serve as a basis for all schools of anesthesia to teach consultation, which remains a difficult exercise for residents. Standardized patient simulation with therefore seems to be an extremely relevant approach for improving the performance of students in CPA, but this aspect will have to be validated by other studies.

## Author’s note

Preliminary data for this study were presented as an oral communication under the reference R422 at the French Society of Anaesthesiology and Critical Care (SFAR) Congress, 22-24 September 2016, Paris.

## Data availability statement

The original contributions presented in the study are included in the article/Supplementary material, further inquiries can be directed to the corresponding author.

## Ethics statement

This research project has received the approval of the Ethics Committee for Non-Interventional Research of Rouen University Hospital (N° E2014-18). The requirement for written informed consent was waived by the Committee. All participants were informed beforehand of the principle of the simulation session and its objectives, as well as the potential interest for training. The presence of an audio-visual system for the real-time retransmission of the session without recording or image processing as well as the presence of professors was reported. All participants have given oral agreement to participate. In the event of occasional image retention, written consent for the image has been signed by each participant.

## Author contributions

EB: Conceptualization, Data curation, Formal analysis, Funding acquisition, Investigation, Methodology, Project administration, Resources, Software, Supervision, Validation, Visualization, Writing – original draft, Writing – review & editing. SF: Conceptualization, Data curation, Formal analysis, Investigation, Methodology, Project administration, Resources, Writing – original draft. AL-S: Validation, Visualization, Writing – review & editing. TW: Methodology, Resources, Writing – review & editing. J-LH: Validation, Writing – review & editing. EA: Validation, Writing – review & editing. BD: Writing – review & editing. VC: Conceptualization, Data curation, Formal analysis, Funding acquisition, Investigation, Methodology, Project administration, Resources, Software, Supervision, Validation, Visualization, Writing – original draft, Writing – review & editing.
